# Safety and Effectiveness of Robotic‐Arm Assisted Total Knee Arthroplasty

**DOI:** 10.1111/os.14008

**Published:** 2024-02-26

**Authors:** Mingcheng Yuan, Tingxian Ling, Qiang Su, Xufeng Wan, Yahao Lai, Zongke Zhou

**Affiliations:** ^1^ Department of Orthopedics West China Hospital of Sichuan University/West China School of Medicine, Sichuan University Chengdu China

**Keywords:** Asian, efficacy, Robotic arm‐assisted total knee arthroplasty, safety, YUANHUA‐TKA system

## Abstract

**Objective:**

We investigated the advantages of robotic arm‐assisted total knee arthroplasty (raTKA) over conventional manual TKA (cmTKA) by comprehensively comparing patients who received raTKA and cmTKA in terms of postoperative pain, function, imaging assessment, and trauma to the body. This study investigated the efficacy and safety of raTKA in patients using the YUANHUA‐TKA system.

**Methods:**

In a prospective, randomized single‐blind trial, 60 patients undergoing primary unilateral TKA from October 2020 to December 2020 were randomly assigned to either raTKA or cmTKA. Clinical evaluation, including the time of osteotomy and prosthesis model testing, the total operation time, the visual analogue scale at rest, VAS in motion, opioid consumption, white blood cell count, neutrophil ratio, erythrocyte sedimentation rate, C‐reactive protein (CRP), passive and active range of motion (pROM, aROM), Western Ontario and McMaster Universities Arthritis Index (WOMAC [stiffness, pain, and function]) score, gait analysis, keen society score (KSS), adverse events, and blood loss were collected by the project nurse, as well as the imaging evaluation, including the lateral tibia component angle (LTC), frontal femoral component angle, frontal tibia component angle (FTC), lateral femoral component angl, and hip–knee–ankle angle (HKA). The student *t*‐test (or the Wilcoxon signed‐rank test) and the χ^2^‐test (or the Fisher exact test) were used to determine differences in categorical variables.

**Results:**

No significant difference was found between the two groups in pain throughout the whole follow‐up period. On the third day postoperatively, the erythrocyte sedimentation rate in the cmTKA group was significantly higher (*p* = 0.02), as well as the CRP (*p* = 0.04). No significant difference was found in the WOMAC stiffnes score or pROM. However, the aROM and the flexion range when walking (FRW) were significantly better in the raTKA group throughout the trial (*p* < 0.05). The KSS at the 1‐month follow‐up and the WOMAC function score at the 1‐year follow‐up were both significantly better in the raTKA group (*p* < 0.05). The HKA and the LTC in the raTKA group closer to the ideal angle, and the difference between the groups was significant (*p* < 0.05). The total operation time of the raTKA group was significantly longer (*p* = 0.001). The intraoperative blood loss had no significant difference in the two groups.

**Conclusion:**

Compared with cmTKA, raTKA with the YUANHUA robot not only avoids extra pain and trauma in patients but promises better functional recovery and improves the accuracy of the prosthesis position and axial alignment reconstruction.

## Graphical abstract

YUANHUA‐TKA is a semi‐active robotic arm system. This robotic arm‐assisted total knee arthroplasty (raTKA) system was authorized for clinical practice by the Chinese National Medical Products Administration in 2020. Compared with MAKO, YUANHUA‐TKA has several advantages. First, the robotic arm of YUANHUA‐TKA has a much smaller volume, which can save space in the operating room, giving the surgeons much more room to operate. Second, the robotic arm of YUANHUA‐TKA has one more axle than MAKO, providing a much more flexible operation. In terms of the cost, YUANHUA‐TKA not only has a much lower acquisition cost but also a lower operating cost because of the rigid driver, rather than the cable driver of MAKO. In this study, we investigated the safety and clinical effectiveness of raTKA using the YUANHUA‐TKA system.

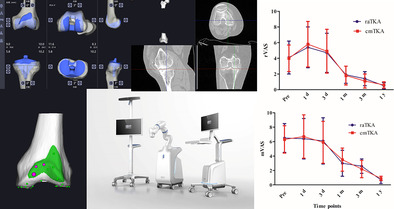

## Introduction

Total knee arthroplasty (TKA) is the most common operation to cure end‐stage knee diseases. Although prosthesis design, surgical techniques, and postoperative rehabilitation programs have advanced significantly over the past two decades, 10%–20% of patients are still dissatisfied following conventional manual (cmTKA) TKA, mainly due to the poor position of the prosthesis, inaccurate recovery of alignment, and the slow recovery of function.^1^ With the rapid development of science and technology in the medical field, the concepts of precision medicine, including intelligent orthopaedics and digital orthopaedics, are gradually capturing the attention of orthopaedists. Computer navigation‐assisted surgery is a typical example of precision medicine.^2^, ^3^ It has been used to improve the accuracy of osteotomy as well ollected from patients in America and Europe, not Asia.

YUANHUA‐TKA is a semi‐active robotic arm system produced by YUANHUA (Shenzhen, China) in 2019. This raTKA system was authorized for clinical practice in 2020 by the Chinese National Medical Products Administration. Compared with MAKO, YUANHUA‐TKA has several advantages. In terms of hardware, first, the robotic arm of YUANHUA‐TKA has a much smaller volume, which can save space in the operating room, giving surgeons much more room to operate. Further, the robotic arm of YUANHUA‐TKA has one more axle than MAKO, which provides a much more flexible operation. In terms of the cost, the YUANHUA‐TKA robot has not only a much lower acquisition cost but also a lower operating cost because of its rigid driver, in contrast to the cable driver of MAKO, which is the design of the robotic arm itself. Mako's robotic arm is joint driven by wires and needs to be calibrated regularly, but the robotic arm of the YUANHUA‐TKA robot is gear driven and has no need to be calibrated regularly.

However, the safety and efficacy of the YUANHUA‐TKA robot has not been investigated or validated previously. We found that studies investigating raTKA preferred to focus on imaging assessment, such as evaluating various angles and axes, rather than investigating the clinical results and the adverse effect simultaneously. Therefore, in this study (i) we investigated the efficacy and safety of raTKA in patients using the YUANHUA‐TKA system; and (ii) we investigated the advantages of raTKA over cmTKA by comparing postoperative pain, function, imaging assessment, and trauma to the body in patients who received raTKA and cmTKA.

## Materials and Methods

This study was registered in the Chinese Clinical Trial Registry (ChiCTR2000031282). The date of registration was March 26, 2020. Approval was obtained from the Clinical Trials and Biomedical Ethics Committee of West China Hospital (HX‐IRB‐AF‐07‐V4.0), and written informed consent was obtained from all patients before participation in this prospective, randomized, single‐blind trial.

### Participants

**FIGURE 1:**
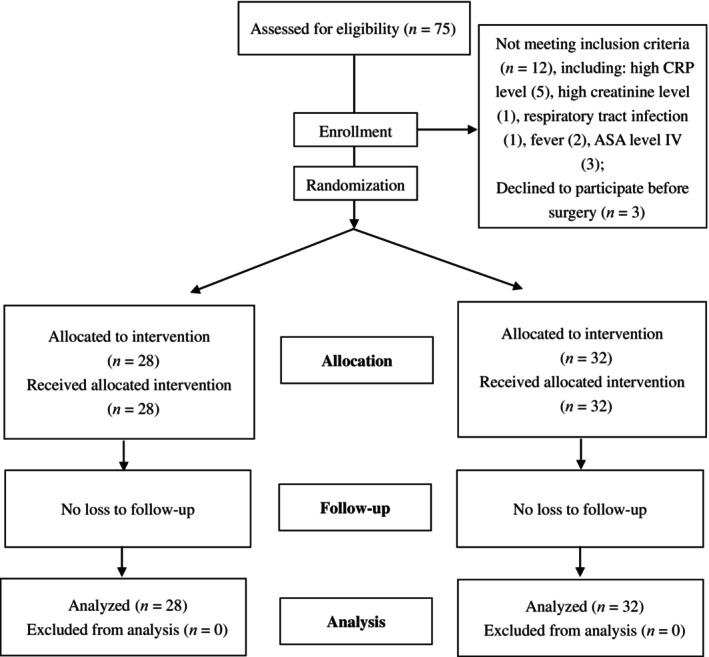
Patient flowchart.

From October 2020 to December 2020, 75 patients who were diagnosed with knee osteoarthritis and scheduled to undergo primary unilateral TKA were eligible for assessment. The inclusion criteria for this study were (i) patients 18–80 years old (no gender limitation); (ii) patients diagnosed with end‐stage knee osteoarthritis and Kellgren–Lawrence grading IV level; (iii) conservative treatment for over 1 year with no significant improvement; and (iv) patients who fully understood the benefits and risks of this study could accept the raTKA treatment and were willing to sign the informed consent form. Patients with the following were excluded: (i) body mass index (BMI) >35 kg/m^2^; (ii) a diagnosis of cardiopulmonary failure; (3) diabetes with poor glycemic control; (4) a history of knee surgery; (5) neuromuscular dysfunction affecting lower limb function; (6) severe coagulopathy; (7) the inability to use a ligament‐sparing prosthesis due to instability; and (8) severe valgus deformity of the knee (Krackow grade III). Finally, 60 patients were enrolled in this study and were divided into two groups using a random number table. There were 28 patients in the raTKA group and 32 patients in the cmTKA group (Figure 1). The preoperative data of the patients are shown in Table 1.

**Table 1:** Baseline data

Parameters	raTKA (*n* = 28)	cmTKA (*n* = 32)	*p*‐value
Age (years)	65.2 (6.4)	65.4 (8.0)	0.88
BMI (kg/m^2^)	27.4 (3.0)	28.4 (3.6)	0.25
Gender (female, *n* [%])	19 (67.9%)	28 (87.5%)	0.07
ASA grade			0.48
I–II	28	28	
III	2	4	
WBC (×10^9/L^)	6.0 (0.8)	6.2 (1.2)	0.46
Neutrophil ratio (*n* [%])	59.1 (5.4)	58.9 (5.8)	0.89
Blood sedimentation (mm/h)	16.4 (7.3)	21.0 (11.0)	0.07
CRP (mg/L)	2.7 (2.2)	3.6 (2.6)	0.15
VAS (score)			
rVAS	4.1 (2.1)	4.0 (1.7)	0.86
mVAS	6.5 (2.0)	6.3 (1.9)	0.81
WOMAC (score)			
Pian	15.9 (4.9)	15.1 (5.1)	0.56
Stiffness	5.4 (2.2)	5.4 (2.3)	0.94
Function	53.2 (10.9)	52.2 (11.2)	0.71
pROM (°)	98.54 (12.89)	96.17 (12.77)	0.48
aROM (°)	97.82 (13.11)	95.13 (12.72)	0.42
KSS (score)	56.79 (11.62)	54.81 (10.72)	0.50

Abbreviations: aROM, active range of motion; ASA, American society of Anesthesiologists; BMI, body mass index; cmTKA, conventional manual total knee arthroplasty; CRP, C‐reactive protein; KSS, keen society score; mVAS, visual analogue scale in motion; pROM, passive range of motion; raTKA, robotic arm‐assisted total knee arthroplasty; rVAS, visual analogue scale at rest; VAS, visual analogue scale; WBC, white blood cell count; WOMAC, Western Ontario and McMaster Universities Osteoarthritis Index.

### Surgical Technique

**FIGURE 2:**
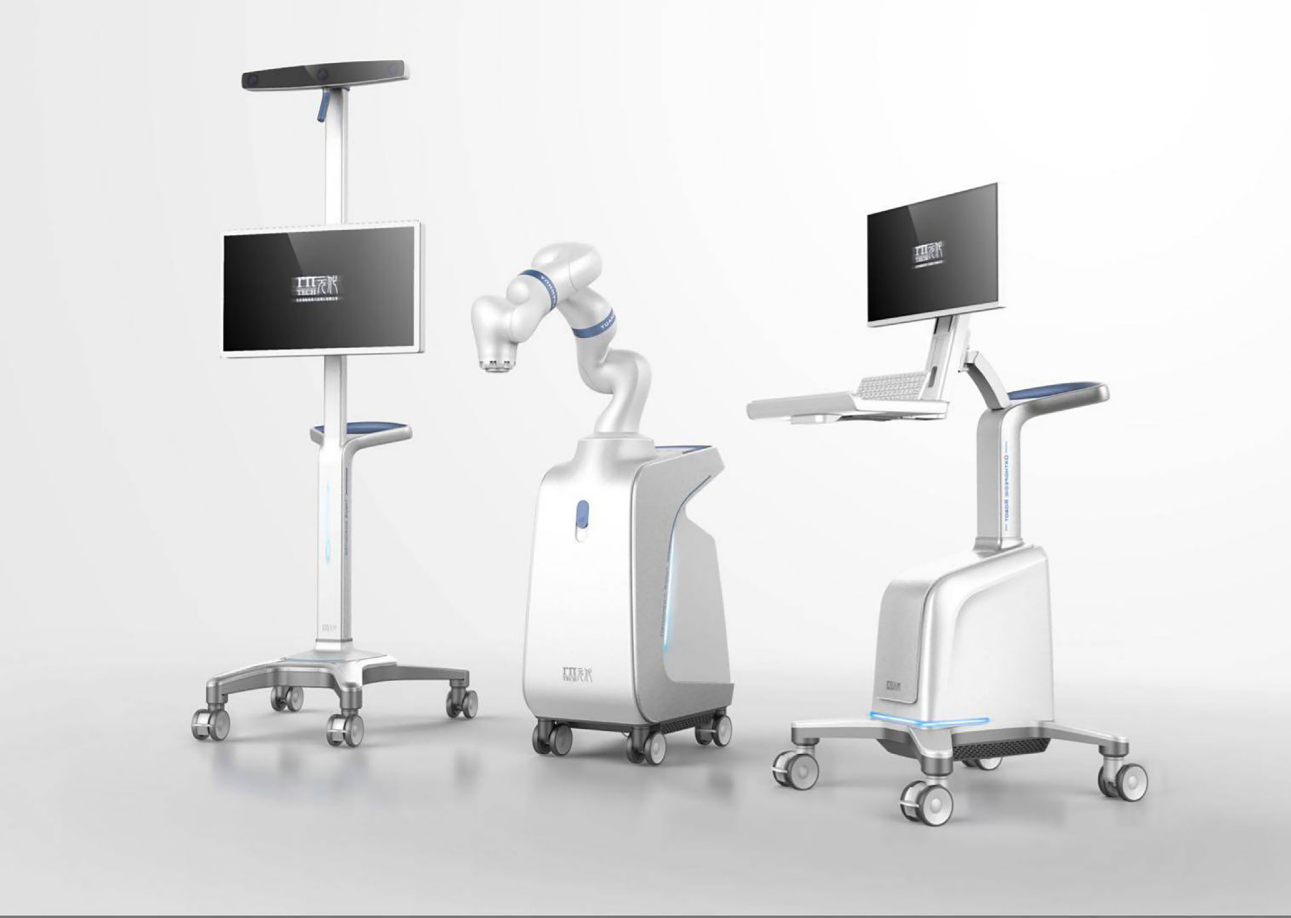
YUANHUA‐TKA robot (Yuanhua Robotics Perception and AI Technologies, Shenzhen, China) is the first semi‐automatic robot orthopaedic surgical robot developed independently in China. It is composed of a navigator, manipulator, and main control console.

All the TKAs were performed by the same surgical team (one surgeon and two assistants). A midline skin incision, a standard medial parapatellar approach, and a cemented posterior stability (PS) total knee system (ZhengTian, Tianjin, China) were used in all patients. All patients received general anesthesia from the same consultant anesthetist. The YUANHUA‐TKA robot (Yuanhua Robotics Perception and AI Technologies, Shenzhen, China) was used in the raTKA group (Figure 2). This is the first semi‐automatic orthopaedic surgical robot developed independently in China. It is composed of a navigator, manipulator, and main control console.

#### Preoperative Planning

**FIGURE 3:**
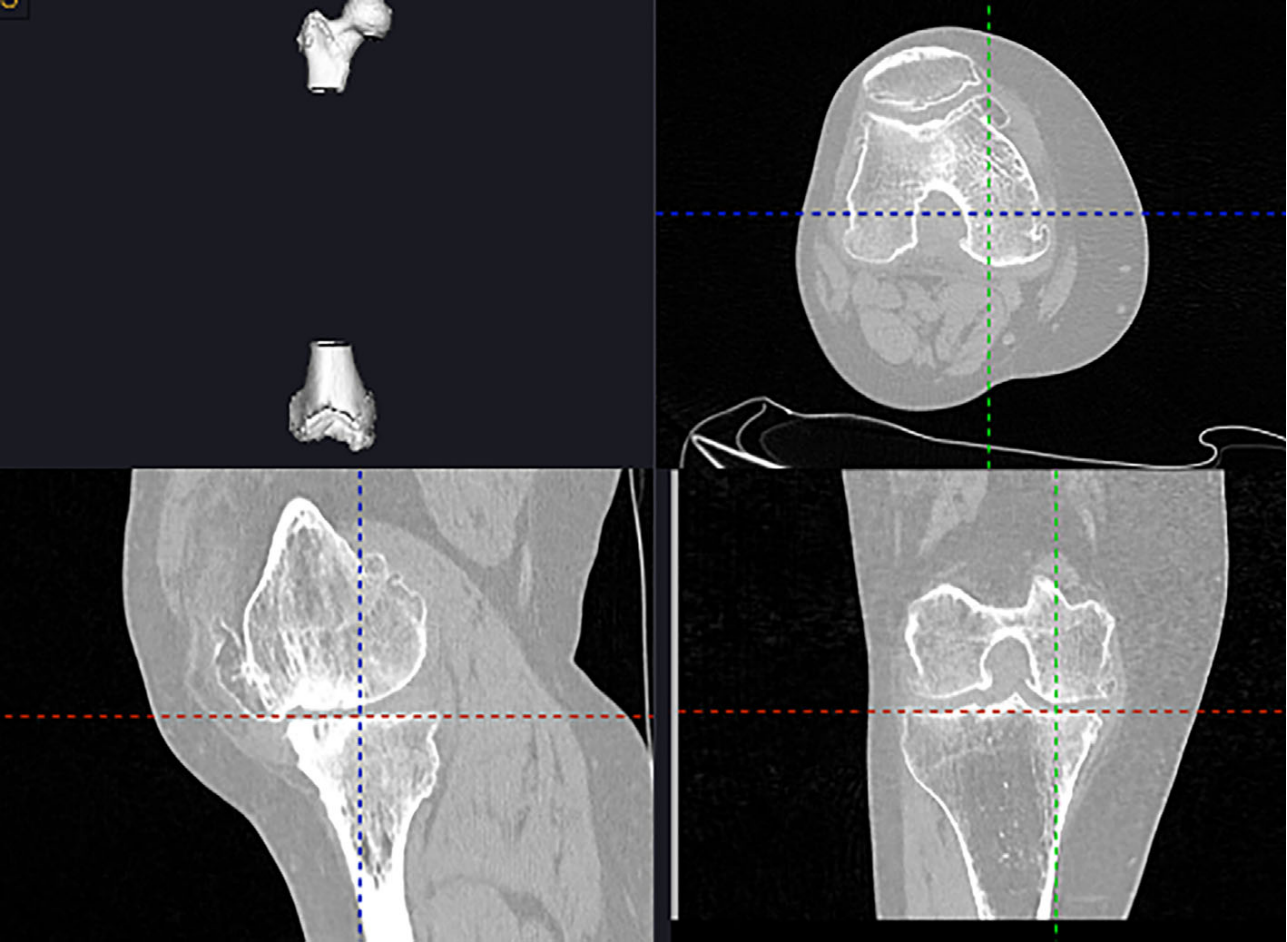
The boundary points were set on the tibia preoperatively.

**FIGURE 4:**
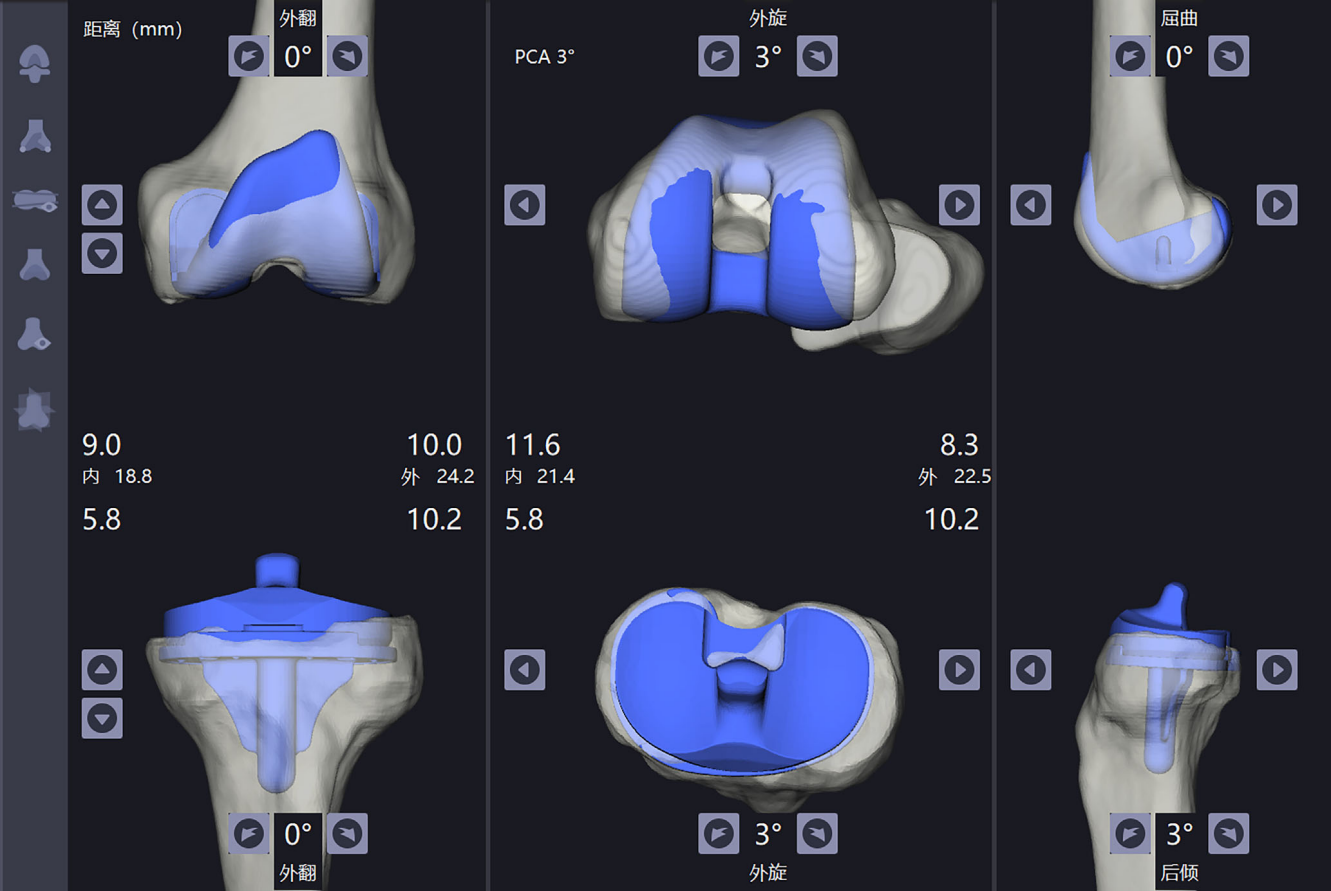
The system generated a preliminary plan. Surgeons could adjust the model of the femur/tibia prosthesis and the spacer as well as the amount of osteotomy individually.

Patients received a CT scan after admission. The scan contained the entire lower limb, including the hip, knee, and ankle. The scan thickness was 3 mm at the hip and ankle and 1 mm at the knee. The CT data were then imported into the YUANHUA‐TKA‐Plan (GuShengYuanHua, Shenzhen, China) preoperative planning software to be automatically segmented and rebuilt into three‐dimensional (3D) models. The boundary points were selected on the 3D model to determine the relevant force lines. The boundary points on the femur were: the acetabular rotation center (the midpoint of the line between the upper and lower margins of the acetabulum), the external/internal epicondyle (vertex of the external/internal epicondyle), the external/medial distal femur (the lowest point of the external/internal condyle on the articular surface of the distal femur), the external/medial posterior femoral condyle (the vertex of the external/medial posterior femoral condyle), and the distal center of the femur (the vertex of the articular surface of the distal femur). The boundary points on the tibia were the external/medial malleolus (the vertex of the external/medial malleolus), the tibial plateau center (the midpoint of the intercondylar fossa), the tibial tuberosity, the center of the posterior cruciate ligament end point (the posterolateral point of the intercondylar fossa), and the external/medial tibia plateau (lowest point of the external/medial tibia plateau) (Figure 3). After choosing the boundary points, the system generated a preliminary plan. Surgeons could adjust the model of the femur/tibia prosthesis and the spacer as well as the amount of osteotomy individually. The adjustment would be updated in real time (Figure 4).

#### Intraoperative Procedure

**FIGURE 5:**
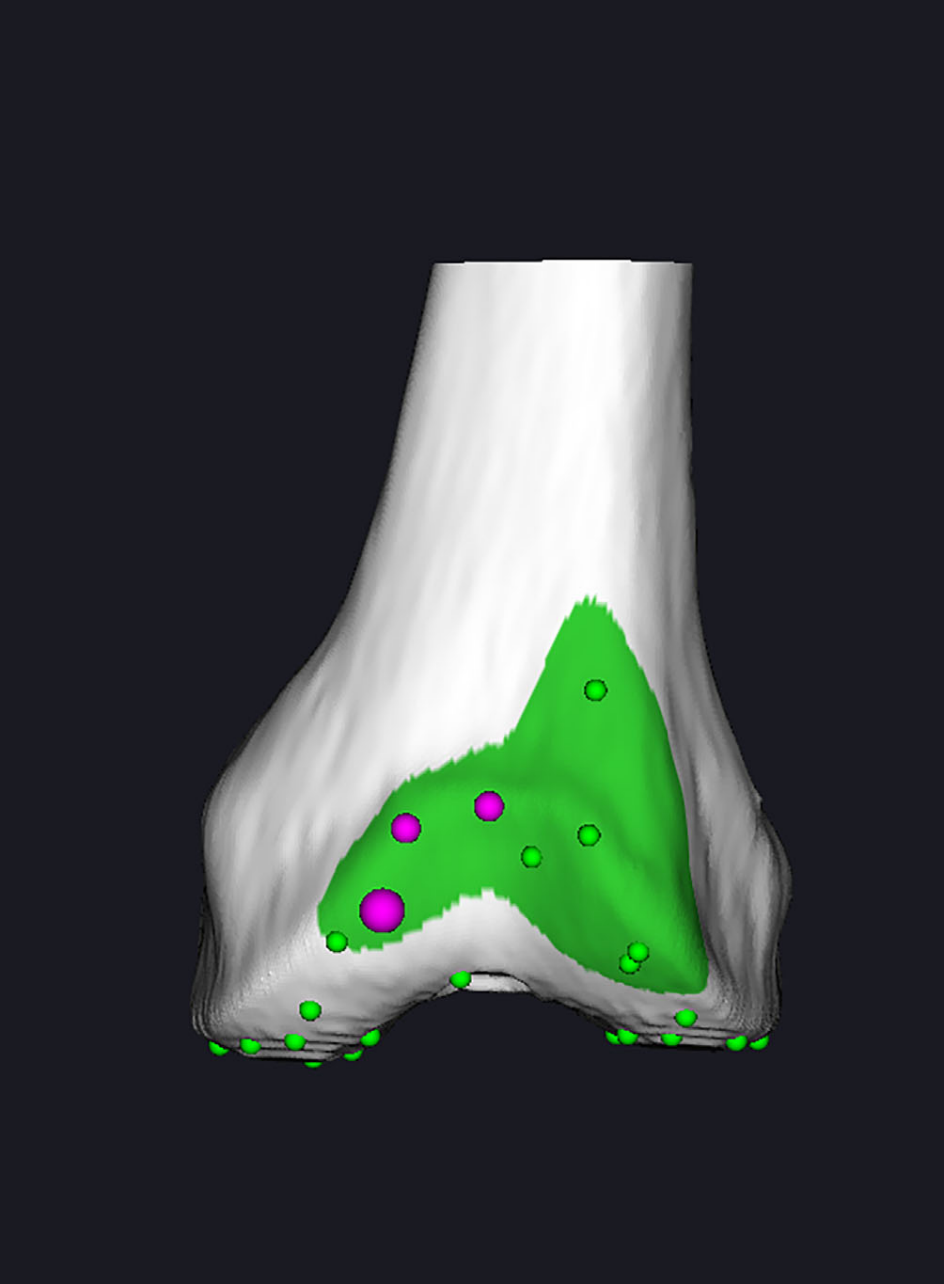
The femur point registration, including five boundary points (purple points) and 30 other points (green points) from the anterior condyle, anterior oblique, distal femoral, and posterior oblique surfaces of the femur.

To install the femoral/tibial tracer, four bone nails must be nailed first, of which two are nailed on the distal femur and two on the proximal tibia. All four nails were 10 cm away from the incision. Then the bone surface registration was started. The 3D model extracted from the CT image was matched with the actual bone of the patient, so we could accurately obtain the spatial position of the patients’ limb and track it in real time. First, the femur point registration was performed, including five boundary points: hip rotation center, external epicondyle, internal epicondyle, distal lateral femur, and distal medial femur. Second, 30 points were collected automatically by the system from the anterior condyle, anterior oblique, distal femoral, and posterior oblique surfaces of the femur. The surgeon used a touch probe to touch 30 points on the bone surface in turn according to the screen. Finally, tibia registration was performed (Figure 5).

Next, the surgeon reassessed the cartilage thickness by putting the probe on the cartilage. The system showed the distance from the top of the probe to the bone surface, which was the thickness of the cartilage. As for the joint space, the navigation system showed the medial and lateral space of the knee in straight and flexed positions. Then, the navigational osteotomy began. The surgeon held the oscillating saw connected to the robotic arm, which could automatically move into position and adjust the angle. During the osteotomy, the surgeon should always watch the screen, which displays the state of osteotomy in real time. The green indicator means insufficient osteotomy and the red means excessive osteotomy, in which case the oscillating saw will automatically cut off the power. If the indicator is white, which means the osteotomy is perfect, no adjustment is required. Moreover, the software has its own safety boundary protection algorithm. Once the saw blade touches the safety boundary, the system stops supplying power to the oscillating saw to ensure the safety of the operation. All the osteotomy steps were conducted manually under the navigation of the system. When the osteotomy was completed, the prosthesis test model was installed. After confirmation that the size of prosthesis is suitable, and the incision was sutured.

#### Blood Management and Thromboembolism Prophylaxis

The patient was given 10 mg/kg of tranexamic acid (TXA) 10 min before the skin incision by intravenous infusion, followed by 10 mg/kg administered by local injection when closing the incision.^4^ Two doses of 10 mg/kg of TXA were separately administered 3 and 6 h after surgery to reduce perioperative blood loss.^5^ Thromboprophylaxis was regularly started 6 h postoperatively with heparin.^6^ The criterion of blood transfusion was set as an Hb level of <70 or 70–100 g/L but with symptomatic anemia (defined as severe mental status changes, palpitations, and/or pallor).

#### Pain Management

Patients received standardized general anesthesia and basic analgesic protocol. From preoperative day 2 to the day before surgery, patients were given celecoxib 200 mg twice a day (one dose after breakfast and the other one after dinner) for preemptive analgesia.^7^ Additionally, an 80‐mL periarticular injection of 0.25% ropivacaine was administered to all patients intraoperatively for local infiltration analgesia. From postoperative day 1, the protocol of oral celecoxib restarted until 3 weeks postoperatively when the patients returned to hospital to have their stitches removed. If acute or resistant pain occurred (VAS > 6), opioids (oral oxycodone or subcutaneous morphine) were used as rescue analgesics.

### Clinical Evaluation

The time of osteotomy and prosthesis model testing as well as the total operation time were recorded from the system intraoperatively. The visual analogue scale at rest (rVAS) and VAS in motion (mVAS) were collected preoperatively and at 1 and 3 days after surgery by a project nurse in the ward. At 1‐month, 3‐month, and 1‐year follow‐up, the items above were also collected by a project nurse from the outpatient department. The opioid consumption was recorded every day from the day of surgery to postoperative day 3 by a project nurse. All opioids, including oral oxycodone and subcutaneous morphine, were converted into morphine equivalents. The inflammatory indicators, including the white blood cell count (WBC), neutrophil ratio, erythrocyte sedimentation rate, and C‐reactive protein (CRP), were recorded preoperatively and at 1‐month, 3‐month, and 1‐year follow‐up. The Western Ontario and McMaster Universities Arthritis Index (WOMAC [stiffness, pain, and function]) score, range of motion (passive and active range of motion [pROM, aROM]), gait analysis, and keen society score (KSS) were recorded preoperatively and at 1‐month, 3‐month, and 1‐year follow‐up. The adverse events were also recorded. Blood loss was calculated from the change in hematocrit using the formula of Nadler et al. and Gross plus the volume transfused.^8^, ^9^

### Imaging Evaluation

**FIGURE 6:**
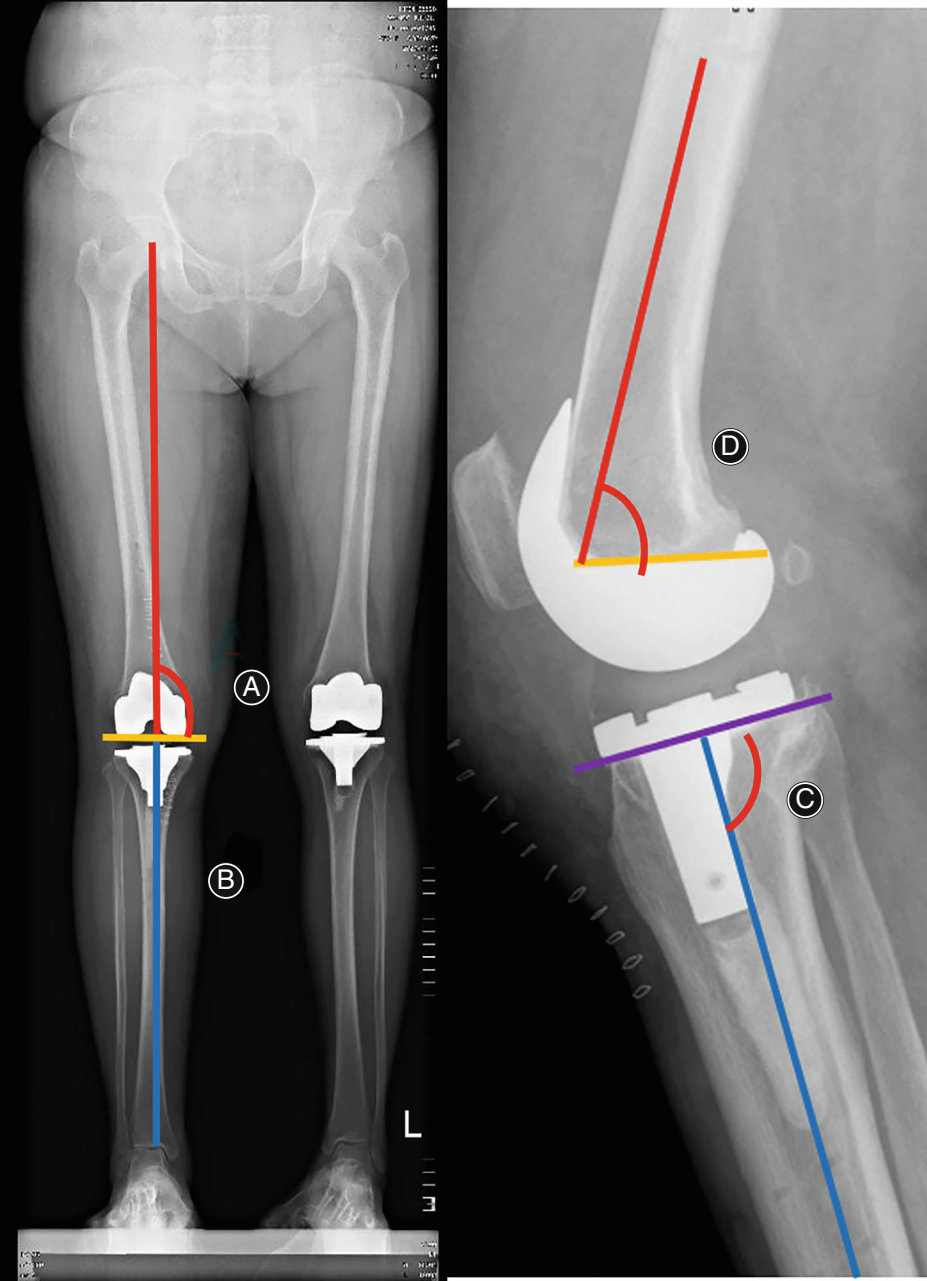
The lateral tibia component angle (LTC) (C), frontal femoral component angle (FFC) (A), frontal tibia component angle (FTC) (B), and lateral femoral component angle (LFC) (D) are shown.

All patients received anteroposterior and lateral projection X‐rays of the knee as well as a full‐length X‐ray examinations of the lower limb. To determine the accuracy of the osteotomy, the position of the prosthesis, and mechanical axis recovery, the following data were measured and recorded from the X‐ray: the lateral tibia component angle (LTC), the frontal femoral component angle (FFC), the frontal tibia component angle (FTC), the lateral femoral component angle (LFC), and the hip–knee–ankle angle (HKA) (Figure 6). To show the difference in the mechanical axis more directly, we used the HKA deviation (HKAD) in place of the HKA.

### Statistical Analysis

Baseline demographics and study outcomes were assessed using measures of central tendency (mean and standard deviation) for quantitative variables (age, BMI, the time of osteotomy and prosthesis model testing, the total operation time, rVAS, mVAS, opioid consumption, WBC, neutrophil ratio, erythrocyte sedimentation rate, CRP, pROM, aROM, WOMAC (stiffness, pain, and function) score, gait analysis, KSS, blood loss, LTC, FFC, FTC, LFC, and HKA) and with percentages for qualitative variables (gender and adverse events). The student *t*‐test or the Wilcoxon signed‐rank test was used to analyze continuous variables, and the χ^2^‐test or Fisher's exact test was used to determine differences in categorical variables. All data analyses were performed using SPSS (version 23.0; IBM). Significance was set at *p* < 0.05.

## Results

The operations in the two groups were successfully completed. All the incisions healed in stage I, and no operation‐related complications occurred.

### Pain Assessment

**FIGURE 7:**
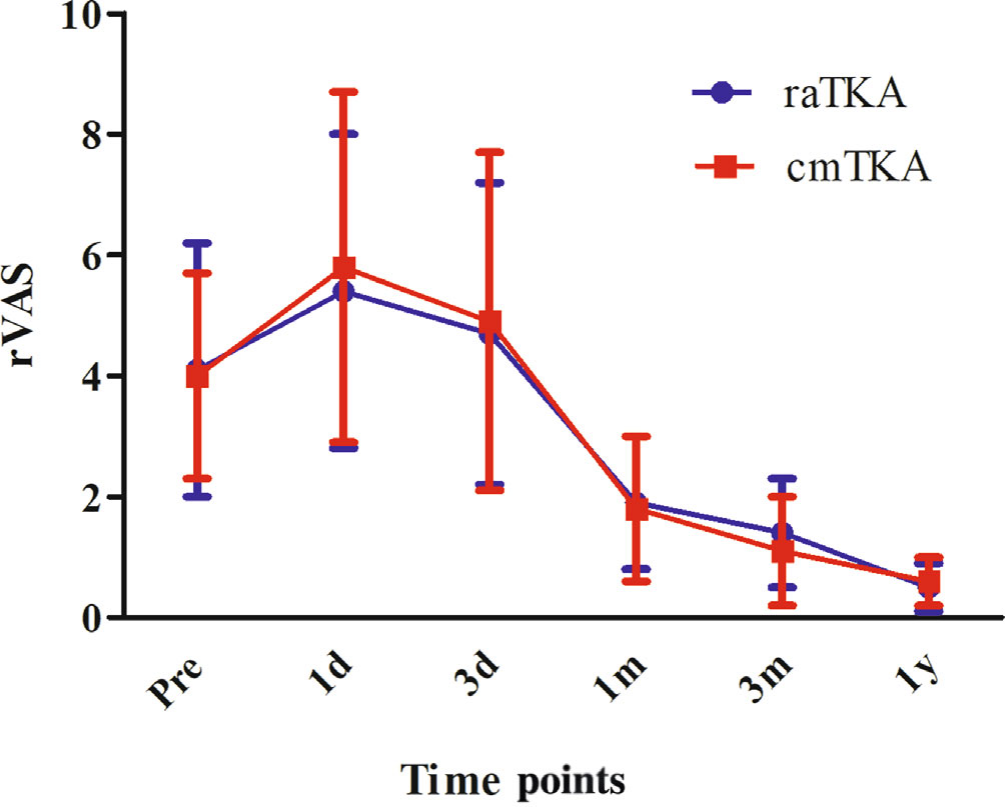
Visual analogue scale at rest (rVAS) at different time points.

**FIGURE 8:**
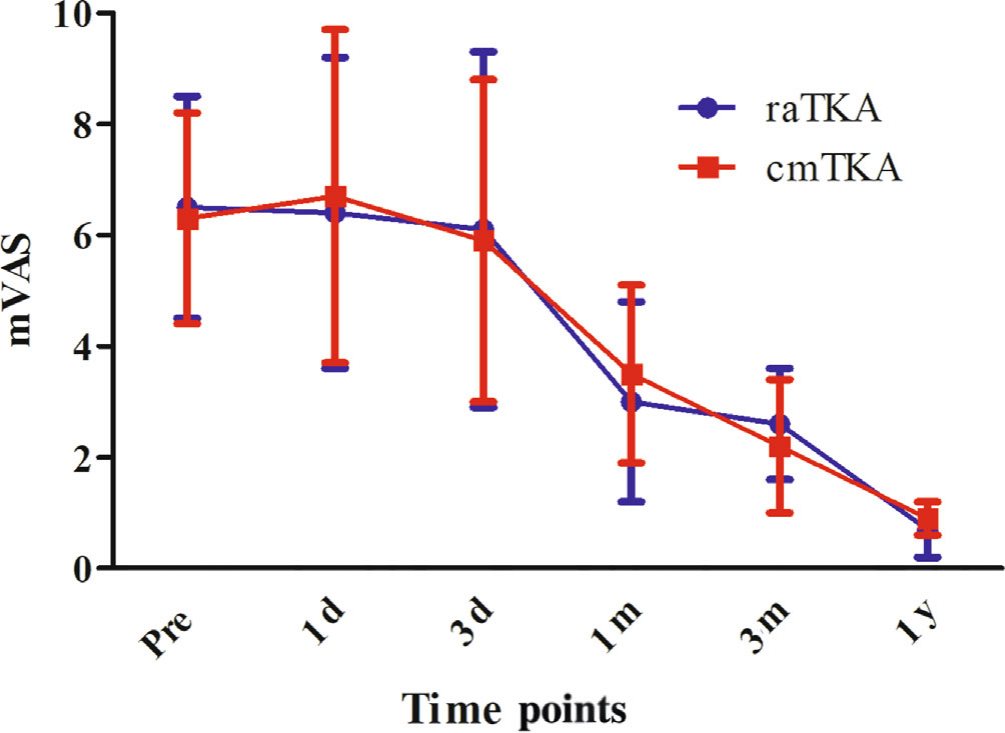
Visual analogue scale in motion (mVAS) at different time points.

No significant difference was found between the two groups in terms of WOMAC pain score, rVAS (Figure 7), mVAS (Figure 8), or opioid consumption throughout the whole perioperative and follow‐up period (*p* > 0.05) (Table 2).

**Table 2:** WOMAC and opioids consumption (mean [SD])

Outcome	raTKA (*n* = 28)	cmTKA (*n* = 32)	*p*‐value
WOMAC (1 month) (score)			
Pain	21.61 (5.04)	22.28 (3.96)	0.56
Stiff	4.29 (2.52)	4.28 (3.08)	0.99
Function	50.57 (13.04)	53.28 (11.72)	0.40
WOMAC (3 months) (score)			
Pain	16.24 (4.13)	15.21 (5.11)	0.39
Stiff	3.24 (2.24)	3.10 (1.98)	0.80
Function	44.04 (10.17)	41.39 (9.76)	0.31
WOMAC (1 year) (score)			
Pain	7.51 (3.17)	7.01 (4.41)	0.89
Stiff	1.24 (1.03)	1.21 (1.28)	0.68
Function	19.04 (8.25)	24.39 (9.01)	0.02*
OC (mg)			
Day 0	15.5 (6.6)	17 (7.2)	0.27
Day 1	22.3 (8.9)	23.7 (11.4)	0.60
Day 3	14.7 (7.0)	16.2 (7.7)	0.44

Abbreviation: OC, opioid consumption.

* Indicates a significant difference.

### Inflammatory Indicators

No significant difference was found between the two groups in WBC or neutrophil ratio. However, on the third day postoperatively, the erythrocyte sedimentation rate in the cmTKA group (59.3 ± 14.1 mm/h) was significantly higher than that of the raTKA group (50.4 ± 15.7 mm/h) (*p* = 0.02), as well as the CRP, which was 79.7 ± 17.4 mg/L in the cmTKA group and 70.4 ± 16.8 mg/L in the raTKA group (*p* = 0.04) (Table 3).

**Table 3:** Inflammatory indicators (mean [SD])

Outcome	raTKA (*n* = 28)	cmTKA (*n* = 32)	*p*‐value
WBC (×10^9/L^)			
Third day	8.1 (2.4)	8.7 (1.9)	0.29
First month	5.6 (1.3)	6.4 (1.9)	0.07
Third month	6.2 (1.7)	6.1 (1.4)	0.80
Neutrophil ratio (*n* [%])			
Third day	75.4 (6.3)	77.3 (7.1)	0.28
First month	61.5 (5.8)	63.5 (6.4)	0.21
Third month	59.7 (6.2)	60.6 (5.5)	0.55
Blood sedimentation (mm/h)			
Third day	50.4 (15.7)	59.3 (14.1)	0.02*
First month	27.1 (9.4)	28.9 (8.5)	0.44
Third month	19.5 (8.3)	22.4 (7.8)	0.17
CRP (mg/L)			
Third day	70.4 (16.8)	79.7 (17.4)	0.04*
First month	6.7 (5.8)	7.3 (6.1)	0.70
Third month	3.6 (3.3)	4.1 (3.7)	0.51

Abbreviations: CRP, C‐reactive protein; WBC, White blood cell count.

* Indicates a significant difference.

### Functional Assessment

No significant difference was found between the two groups in terms of the WOMAC stiffness score (Table 2) or pROM (Table 4). However, the aROM and the flexion range when walking (FRW) were significantly better in the raTKA group throughout the trial (*p* < 0.05) (Table 4). The KSS at the 1‐month follow‐up (Table 4) and the WOMAC function score at the 1‐year follow‐up (Table 2) were significantly better in the raTKA group (*p* < 0.05).

**Table 4:** Functional assessment (mean [SD]).

Outcome	raTKA (*n* = 28)	cmTKA (*n* = 32)	*p*‐value
pROM (°)			
First month	104.82 (9.08)	102.03 (6.70)	0.18
Third month	107.10 (10.97)	105.00 (10.91)	0.52
1 year	115.10 (8.73)	114.00 (6.34)	0.76
aROM (°)			
First month	101.82 (7.10)	97.03 (10.5)	0.046*
Third month	105.10 (6.84)	101.07 (7.23)	0.03*
1 year	110.82 (7.42)	106.05 (7.88)	0.02*
KSS (score)			
First month	66.21 (11.76)	60.72 (9.13)	0.048*
Third month	80.42 (12.37)	77.21 (11.35)	0.30
1 year	91.48 (7.62)	90.21 (8.22)	0.60
FRW (°)			
First month	40.2 (10.71)	34.5 (9.63)	0.03*
Third month	46.21 (9.66)	38.41 (10.94)	0.003*
1 year	48.73 (8.10)	43.17 (8.55)	0.01*

Abbreviations: aROM, active range of motion; FRW, flexion range when walking; KSS, keen society score; pROM, passive range of motion.

* Indicates a significant difference.

### Imaging Evaluation

Until the 1‐year follow‐up, no case of dislocation or loosening occurred in either group. The HKA, LTC, FFC, FTC, and LFC were exactly the same at different time points, so we only listed the data at the 1‐year follow‐up (Table 5). The HKA and the LTC in the raTKA group were more closed to the ideal angle (HKA is the “hip–knee–ankle angle,” so the ideal HKA is 180°; LTC is the co‐angle of the posterior slope angle of the tibial plateau [ideal angle is 3°], so the ideal LTC is 87°), and the difference between groups was significant (*p* < 0.05).

**Table 5:** Imaging evaluation (mean [SD]).

Outcome	raTKA (*n* = 28)	cmTKA (*n* = 32)	*p*‐value
HKA deviation (°)	1.3 (0.6)	2.2 (1.7)	0.014*
LTC (°)	2.8 (1.5)	5.3 (2.8)	<0.001*
LFC (°)	1.9 (1.9)	2.5 (2.7)	0.298
FTC (°)	89.1 (0.5)	88.7 (1.1)	0.134
FFC (°)	89.0 (1.0)	88.8 (1.1)	0.369

Abbreviations: FFC, frontal femoral component angle; FTC, frontal tibia component angle; HKA, hip‐knee‐ankle angle; LFC, lateral femoral component angle; LTC, lateral tibia component angle.

* Indicates a significant difference.

### Other Evaluation

The total operation time of the raTKA group (108.7 ± 17.3 min) was significantly longer than that of the cmTKA group (95.4 ± 11.2 min) (*p* = 0.001), but the times of the osteotomy and prosthesis model testing were significantly shorter in the raTKA group after the surgeon's 14th raTKA operation (osteotomy: 11.9 ± 4.4 vs. 16 ± 0.9 min *p* < 0.001; prosthesis model testing: 19.9 ± 2.8 vs. 22.5 ± 2.3 min *p* < 0.001), which demonstrated the precision of the navigational osteotomy without extra try and trim. The intraoperative blood loss in the raTKA group (272.8 ± 57.1 mL) had no significant difference from the cmTKA group (246.2 ± 52.4 mL) (*p* = 0.071) (Table 6). There were also no cases of periprosthetic fracture, infection, joint stiffness, or secondary admission for any reason in either group.

**Table 6:** Other indexes (mean [SD]).

Outcome	raTKA (*n* = 28)	cmTKA (*n* = 32)	*p*‐value
Total operation time (min)	108.7 (17.3)	95.4 (11.2)	0.001*
Time of osteotomy (min)	11.9 (4.4)	16 (0.9)	<0.001*
Time of the prosthesis model testing (min)	19.9 (2.8)	22.5 (2.3)	<0.001*
Intraoperative blood loss (mL)	272.8 (57.1)	246.2 (52.4)	0.071

* Indicates a significant difference.

## Discussion

In this study, we found that compared with cmTKA, raTKA with the YUANHUA‐TKA robot would not only avoid extra pain or trauma in patients but could promise better functional recovery and improve the accuracy of the prosthesis position and axial alignment reconstruction.

Compared with other robots, the robotic arm of YUANHUA‐TKA has one more axle, which means there is no need to switch the end of the oscillating saw from a right angle to a sagittal shape when cutting the distal femur and the posterior inclined surface. Therefore, there is no need to re‐calibrate the saw, which can increase the continuity of the operation.

### Postoperative Pain

We found that both the rVAS and the mVAS in the two groups perioperatively and at follow‐up had no significant difference between the two groups at any time point; nor did the OC or the WOMAC pain score, which was consist with the 2021 meta‐analysis by Kort N et al.^10^ They investigated 4567 raTKAs and 5966 cmTKAs in seven studies and found that the robotic assistance indeed improved clinical scores, including WOMAC function score and KSS, but no significant difference was found in pain scores, like VAS or WOMAC pain score, which was consistent with the study of Onggo et al.^11^ comparing 2234 rTKA and 4300 cmTKA. The causes of patients’ pain were mainly from two aspects: macroscopically, postoperative pain emerged from the injury of deep tissue caused by surgical incision and surgical approach; microscopically, pain was mainly from the local inflammatory response caused by surgical stimulation. The inflammatory mediators released and then stimulated the local nerve endings in the human body, causing pain reflex.^12^ In this trial, both the raTKA and cmTKA used the same midline skin incision and the standard medial parapatellar approach, which meant the damage to the soft tissue such as skin and muscle was almost the same. Even though in the cmTKA group surgeons needed to dislocate the tibial forward and turn the patella outward to operate, many studies have demonstrated that no extra pain is caused in this procedure.^13^, ^14^, ^15^ We also recorded the inflammatory indicators postoperatively and found that the erythrocyte sedimentation rate and CRP on postoperative day 3 in the raTKA group were significantly lower. However, the pain scores had no significant difference between the groups. We speculated that although the inflammatory indicators in the body were connected to the pain to some extent, the difference was not large enough to affect the patients. The similar opioid consumption of the two groups suggested that the raTKA would not cause extra acute or serious pain postoperatively.

### Function Recovery

In terms of the recovery of function, we discovered something interesting: Although the pROM was approximately the same between the two groups throughout the trial (*p* > 0.05), the aROM in patients who received raTKA was always better than in those in the cmTKA group (*p* < 0.05). Further, the flexion range when walking recorded from the gait analysis was also significantly better in the raTKA group, indicating that in the raTKA group, active flexion and extension of patients’ knee were more relaxed; walking and other daily activities were freer. There are two main potential reasons for the better active motion in the raTKA group. First, the intraoperative handle of the patella in the cmTKA group mentioned above might generate an extra pulling force on the quadriceps femoris, which increased torsion stress to the quadriceps femoris and, therefore, decreased the active range of motion and the freedom to do daily activities in some patients.^16^ However, intraoperative traction did not cause any real damage to the quadriceps femoris in either group, so the pROM, which was recorded under an external force, was not significantly different between the groups at all. Moreover, it was found in our study that patients’ knees in the cmTKA group were generally a little more swollen. It was reported that the pulling of muscle might promote the activation of an inflammatory response.^17^ We suspected that the pulling of the quadriceps femoris in the cmTKA group might account for the higher inflammatory indicators in the early postoperative period in the cmTKA group, as recorded,^18^ and in the meanwhile promote the swelling of the knee in the cmTKA group. When the limb was swollen, there was little space around the knee for patients to flex totally and actively without an external force. Besides aROM, the KSS at 1‐month follow‐up and the WOMAC function score 1 year postoperatively were also better in the patients who received raTKA, which further confirmed a relatively better functional recovery with raTKA. This was consistent with the study of Kort et al.,^10^ who found that the robotic assistance could improve clinical scores, including the WOMAC function score and KSS, more than manual TKA could.

### Precise TKA

It was reported that the probable osteotomy deviation in cmTKA might cause imbalance of the medial and lateral gap of the joint and unsatisfactory recovery of the mechanical axis of the lower limb, which would lead to instability and further increase the incidence of polyethylene liner wear, prosthesis loosening and subsidence, and, finally, reduce the long‐term survival rate of the prosthesis.^19^ Therefore, robotic assistance in TKA has gradually captured the attention of orthopaedic surgeons. The surgical robots integrated the computer navigation, synchronous screen, and mechanical arm. Before the surgery, it could calculate the thickness and angle of osteotomy at different positions automatically according to the plan, which was designed by the surgeons according to the CT scan. They took into consideration the thickness of the osteotomy under the prosthesis, the angle of the distal‐femoral valgus, and the lateral femoral condyle osteotomy. With such accurate calculations, the tibial PS prosthesis could obtain an tibial prosthesis tilt angle (3°) on the sagittal plane. A perfect restoration of the mechanical axis of the lower limb could also be achieved. Marchand et al.^20^ measured the mechanical axis of the lower limb in 330 patients who received raTKA and found that although 64% of patients had varus deformity of more than 3° and 11% had valgus deformity of more than 3°, all patients’ mechanical axes of the lower limb were finally corrected to the near neutral position after surgery (±2°). In this study, all the mechanical axes of the lower limb and the tibial PS prosthesis tilt angles in the RATKA group were within the ideal range (mechanical axis of the lower limb: 0° ± 2°; tibial prosthesis tilt angle: 3° ± 1.5°) and significantly better than those in the cmTKA group (*p* < 0.05). Although other angles, including LFC, FTC, and FFC, had no significant difference between the two groups, those angles in the raTKA group had a lower standard deviation, which suggested that robotic assistance in TKA could make the results of osteotomy more stable around the ideal angle with a relatively smaller deviation.

### Safety Evaluation

As for the adverse events, no significant difference was found in either the perioperative period or during the 1‐year follow‐up. The intraoperative blood loss and perioperative pain had no significant difference between the two groups, suggesting that although raTKA had a relatively longer operation time and two more small incisions were needed for the bone screws to be inserted into the femur and the tibia for installing the tracers, no extra trauma or pain feeling were caused to the patients. The incidence of nausea, vomiting, coughing, insomnia, and fat liquefaction of wounds also had no significant difference between the groups. This might account for the enhanced recovery after surgery (ERAS) strategy in our center, which is a verified perioperative management principle in TKA.^6^ There were also no cases of periprosthetic fracture, periprosthetic infection, joint stiffness, or secondary admission in either group. Even though no case of dislocation or loosening occurred in either group, the mechanical axis of the lower limb and tibial PS prosthesis tilt angle in the RATKA group were still better than those in cmTKA group. However, the prosthesis survival rate still requires long‐term follow‐up.

### Learning Curve

Robotic arm‐assisted TKA, as a newly emerged technology, requires surgeons to undergo the process of learning and becoming proficient in performing the procedure. Although compared with cmTKA, raTKA included extra time for femur registration, tibia registration, and oscillating saw registration, as the surgeons became more familiar with the equipment, the time of femur registration, femur osteotomy, osteotomy adjustment, prosthesis testing, prosthesis implantation, and the total time of surgery would gradually decrease. Finally, the total operation time of raTKA could be about the same as the cmTKA, even shorter. In this study, we found that the learning curve of raTKA was seven to 14 cases, which was consistent with Marchand et al. (2022), reporting 11 cases as the learning curve using the MAKO robot.^21^

### Strengths and Limitations

Some strengths of this study should be mentioned. First, the sample size of this study was relatively large compared to other robotic arm‐assisted TKA research in Asia. Second, we investigated the efficacy and safety of raTKA in patients in several aspects, including postoperative pain, function, imaging assessment, and trauma to the body, using the YUANHUA‐TKA system. Third, we not only focused on the postoperative pain assessment but also identified the possible causes behind the pain.

However, certain limitations of the present study should be mentioned. First, our test of pain might be not sensitive enough to detect the delicate difference in feeling in patients, and we did not record the thigh circumference to evaluate the swelling quantitatively. Second, this study was based on the data collected from the single center of our hospital, which only represents the results for Southwest China. Data from more centers are needed to further demonstrate the safety and efficacy of raTKA with the YUANHUA robot.

## Conclusion

Compared with cmTKA, raTKA with the YUANHUA‐TKA robot would not only avoid extra pain or trauma in patients but could promise better functional recovery and improve the accuracy of the prosthesis position and axial alignment reconstruction.

## Funding Information

This work was supported by the 1.3.5 Project for Disciplines of Excellence, West China Hospital, Sichuan University (No. 2023HXFH012) and the National Natural Science Foundation of China (No. U22A20280).

## Conflict of Interest Statement

The authors have no competing interests to declare.

## Author Contributions

This study was conducted under the guidance of ZK‐Z. The article was written by MC‐Y and TX‐L. The clinical assessment was done by Q‐S and XF‐W. The statistical analysis was performed by YH‐L. All authors read and approved the final manuscript.

## Authorship Declaration

All authors listed above meet the authorship criteria according to the latest guidelines of the International Committee of Medical Journal Editors and are in agreement with the manuscript.
